# Validity and reproducibility of an interviewer-administered food frequency questionnaire for healthy French-Canadian men and women

**DOI:** 10.1186/1475-2891-3-13

**Published:** 2004-09-13

**Authors:** Julie Goulet, Geneviève Nadeau, Annie Lapointe, Benoît Lamarche, Simone Lemieux

**Affiliations:** 1 Institute of Nutraceuticals and Functional Foods, Laval University, Québec, Canada

**Keywords:** Food frequency questionnaire, validation, reproducibility, 3-day food record

## Abstract

**Objective:**

To evaluate the validity (study 1) and the reproducibility (study 2) of an interviewer-administered food frequency questionnaire (FFQ).

**Method:**

The FFQ was designed at Laval University and contains 91 items and 33 subquestions. *Study 1*: The FFQ was compared against a 3-day food record (2 week-days and 1 weekend-day), at week 0, 6 and 12 of a nutritional intervention. *Study 2*: In order to evaluate the reproducibility of the FFQ, 2 registered dietitians administered the FFQ 4-weeks apart among subjects who were not part of the nutritional intervention.

**Results:**

*Study 1*: Mean values for intake of most nutrients assessed by the FFQ and by the 3-day food record were not statistically different. Energy-adjusted correlation coefficients for major macronutrients ranged from 0.36 for proteins to 0.60 for carbohydrates (p ≤ 0.01). Agreement analysis revealed that on average, 35% of the subjects were classified in the same quartile when nutrients were assessed by either the 3-day food record or the FFQ. *Study 2*: Significant associations were observed between dietary measurements derived from the two FFQs administered 4 weeks apart. Correlation coefficients for the reproducibility of macronutrients ranged from 0.66 for carbohydrates to 0.83 for lipids after energy adjustment. On average, 46% of the subjects were classified in the same quartile when nutrient intakes were assessed by either FFQ.

**Conclusion:**

These data indicated that the FFQ developed has a good validity and is reproducible.

## Background

There is increasing evidence that nutrients may be important in the development of chronic diseases such as coronary heart disease (CHD) and type 2 diabetes. In the late 60s, the Mediterranean diet became a topic of interest primarily because of results of the Seven Countries Study, which demonstrated that the 15-y mortality rate from CHD in Southern Europe, was two to threefold lower than in Northern Europe or United States [1]. More recently, results from The Lyon Diet Heart Study showed that a Mediterranean alpha-linolenic acid-rich diet prevented the recurrence of cardiovascular events more than did the usual prudent Western diet in men [2-4]. Reliable instruments for diet measurements are necessary to identify which components of the Mediterranean diet are the best candidates to explain, in part, such a protective effect.

Accurate assessment of dietary intakes, when based on self-report in free-living populations poses significant scientific challenges. All standard dietary assessment methods including food records, dietary recalls and list-type methods such as food frequency questionnaires (FFQ), are subjected to considerable error and bias, and none of these can be considered as a 'gold standard' measure [5]. FFQ has become a common way to estimate usual food intake because it usually requires less than thirty minutes to complete [6]. It also imposes less burden on subjects than most of the other dietary assessment methods. However, disadvantages of the FFQ have been identified. In fact, it may be difficult cognitive task for respondent to recall frequencies of intakes over a given period of time. Also, the precision in quantifying intakes is not possible with a FFQ. Dietary habits vary not only from country to country but also from region to region. Specific FFQs must be validated to assess nutritional habits conducted in geographically and/or culturally distinct regions [6]. It is also important in nutritional intervention to consider the sensitivity of the method over the duration of a study, especially in study that is testing the effects of dietary changes [7].

In Québec, no validated FFQ was adapted for the needs of our nutritional intervention design. In fact, in the context of our nutritional intervention we wanted to use a FFQ to evaluate a Mediterranean food pattern in a North-American context that would contain foods available in Québec and also foods characteristic of the Mediterranean diet. In order to improve the precision in quantifying reported intakes we decided to use and interviewer-administered FFQ to facilitate the determination of portion size using food models. Thus, we decided to design an interviewer-administered FFQ to assess the dietary changes among a French-Canadian population in a nutritional intervention promoting the Mediterranean food pattern. The first purpose of the present study was therefore to test the validity of this interviewer-administered FFQ. In order to reach this objective, nutrient intakes derived from the FFQ were compared to intakes obtained by the 3-day food record. Comparisons were performed for baseline as well as for post-intervention values. This allowed us to determine whether our FFQ would permit to identify similar changes in nutrient intake in response to the intervention as the ones measured by the 3-day food record. As a second objective, we also wanted to estimate the short-term reproducibility of this FFQ in a control group who did not receive the nutritional intervention.

## Subjects and methods

### Subjects

This paper reports results of two studies: 1) a study on validity of the FFQ tested against a 3-day food record during a nutritional intervention program promoting the Mediterranean food pattern and 2) a study on reproducibility of the FFQ in a control population.

For the validation study, women from the Québec City metropolitan area were recruited through the Laval University newspaper during the summer of 2001. Women included in the study were aged between 30 to 65 years [8]. To be eligible, women had to be free from metabolic disorders requiring treatment, to have stable body weight for at least 3 months prior to the start of the study and to be in charge of food purchases and meal preparation most of the time. One hundred and twenty six women were invited to a screening visit for an evaluation of their food habits. Among this initial group of women, 94 were found to be eligible according to the above criteria and 77 women agreed to take part to the study. Three women left the study during the 12-week intervention for personal reasons. One participant did not complete the FFQ at week 12 and 2 other participants did not complete all three food records. Therefore, 71 women were included for the FFQ validation analysis.

For the reproducibility study, 20 men and 19 women from the Québec City metropolitan area were also recruited through the Laval University newspaper during the summer of 2002. Men and women included in the study were aged between 25 to 70 years. Three men and 4 women did not complete the second FFQ for personal reasons. Therefore, 17 men and 15 women were included for the reproducibility analyses. The study was approved by the Ethics Committees of Laval University.

### Food frequency questionnaire

The interviewer-administered FFQ developed inquired on the food habits during the last month and is based on typical food items, which are available in Québec. It contains 91 items among which 27 had between 1 and 3 subquestions. The FFQ was structured to reflect Quebecers' food habits and food items were listed in food groups (vegetables; fruits; legumes, nuts and seeds; cereals and grain products; milk and dairy products; meat/processed meat; poultry; fish; eggs; sweets; oils and fats; fast foods and drinks). Because of the nature of our nutritional intervention, our FFQ was designed to make sure to document with enough details consumption of typical items of the Mediterranean diet such as type of oils, whole grain products and legumes. The 30-minutes FFQ was administered face-to-face by one of the 3 registered dietitians involved in the study. During the interview, the dietitians used food models for a better estimation of the real portion consumed by the subject. Participants were questioned about frequency of intake for different foods during the last month and were asked to report the frequency of these intakes in terms of day, week or month. The subquestions allowed a better definition of the food items consumed. For example, following the question "How often do you eat yogurt?" subjects were asked about the fat percentage and brand of the yogurt consumed. An open question at the end of the FFQ allowed individuals to report any other frequency eaten foods not listed in the FFQ and provide details about usual recipes used in order to quantify better intakes of individual food items.

### Cut-off value to evaluate energy intake

Estimates of basal metabolic rate (BMR) were calculated from the Harris-Benedict formulas based on height, weight, age and sex [9]. Energy intake reported from FFQs and from 3-day food records were compared with estimates of BMR to calculate the number of participants who may have underreported their energy intake [10]. It is suggested that a ratio between energy intake and estimate BMR of less than 1.35 might not represent long term habitual intake in a non dieting population [10].

### Study 1: Validation

#### Intervention and FFQ

The methodology of the nutritional intervention has been described previously [8]. Briefly, the study was conducted in 2 phases. Each phase was conducted using a similar 12-week intervention design. The FFQ was administered at screening (t = 0) and then at weeks 6 and 12. The intervention included 2 group sessions. Individual sessions took place during the 1^st^, the 6^th ^and 12^th ^weeks of the intervention in order to evaluate the changes and to select further objectives for increasing the adherence to the Mediterranean food pattern. Three registered dietitians were trained to provide a standardized intervention.

#### 3-day food record

Each participant completed a 3-day food record, 2-week days and 1-weekend day, at week 0, 6 and 12. At screening of the nutritional intervention, a dietitian provided 15 minutes of instructions to each participant on how to complete the food records. Written copies of record examples were provided to each subject. Also, participants were encouraged to consume usual amounts of typical foods and drinks for the completion of the food record. Participants were not required to weight foods but were asked to measure the volume of foods consumed with household measurements (cups, tablespoons) or to indicate the weight of commercial products when it was possible to assess portion sizes. The food record included a section for recording information recipes. After completing the food record, participants met with the dietitian to review all the information for record accuracy and completeness and portion size of individual items on the food record were reviewed when needed by using food models.

#### Anthropometry

At weeks 0, 6 and 12, body weight and height were measured according to the procedures recommended at the Airlie Conference on the Standardization of anthropometric measurements [11] and body mass index (BMI) was calculated.

### Study 2: Reproducibility

#### Study design

The 32 participants of the reproducibility study were distributed into two groups. In the first group, dietitian #1 administered the FFQ and 4 weeks later dietitian #2 administered the FFQ for the second time. Inversely, in the second group of participants, dietitian #2 administered the first FFQ and 4 weeks later dietitian #1 administered the FFQ for the second time. An interval of one month was chosen to reduce any training effect and memory influence of the method. Both dietitians were taught to use the FFQ similarly, using the same examples of portion size, and asking similar questions.

#### Anthropometry

At the first visit, body weight and height were measured according to standard procedures [11] and BMI was calculated.

### Analysis

#### Nutritional analysis

Evaluation of nutrient intakes derived from the FFQs and food records was performed using the Nutrition Data System for Research (NDS-R) software version 4.03, developed by the Nutrition Coordination Center, University of Minnesota, Minneapolis, MN, Food and Nutrient Database 31, released in November 2000 [12]. This database includes more than 16 000 food items for which the complete nutritional value of 112 nutrients is included. For the purpose of our study, intakes of selected nutrients susceptible to affect the CHD risk profile were analysed: energy, proteins, carbohydrates (CHO), lipids, saturated fatty acids (SFA), monounsaturated fatty acids (MUFA), polyunsaturated fatty acids (PUFA), trans fatty acids, eicosapentaenoic acid (EPA), docosahexaenoic acid (DHA), total as well as insoluble and soluble dietary fibers, vitamin C, folate, iron and calcium. Intakes of vitamin and mineral supplements were not included in the present analysis, which focused on dietary nutrients only.

#### Statistical analysis

In the validation study, means and standard deviations for nutrient intakes were calculated from the FFQs and from the food records. Student t-tests were performed to determine the differences between nutrient intakes assessed by the FFQ and by the 3-day dietary record. Since many variables were not normally distributed, Spearman correlations were used to put into relationship nutrient intakes from FFQ with those from the food record. Student t-tests were also performed to determine the differences between changes in nutrient intakes in response to the nutritional intervention assessed by the FFQ and by the 3-day dietary record. Agreement analyses were performed to verify the concordance of different nutritional variables among quartiles of the distribution between FFQ and the 3-day food record. In the reproducibility study, Student t test was performed to assess the differences between both FFQs. Spearman correlations were used to put into relationship the nutrients reported in the first and the second FFQ. Agreement analyses were assessed to verify the degree of concordance in classifying subjects among quartiles of the distribution between both FFQs. For both studies, the dietary variables were log transformed when necessary to achieve a normal distribution, and the formula log(x + 1) was used for alcohol because some subjects had a value of 0 g for alcohol intake. In addition, to make the comparisons based on absolute nutrient intakes, correlations were also made using energy-adjusted variables. Adjustment for total energy intake was achieved by using the residual method proposed by Willett and Stampfer [13]. Residuals are computed from regression models, with total energy intake as the independent variable and nutrient intakes as the dependent variable. Values were considered as being very well correlated for correlations ranging between 0.7 to 0.9, well correlated for correlations ranging between 0.5 to 0.7 and moderately well correlated for correlations between 0.3 to 0.5, as suggested by Rimm et al [14]. Because age and BMI may have influenced the manner in which subjects answered the FFQ or completed the 3-day food record, partial correlations for age and BMI were also computed. Also, Student t-tests were performed to verify variation in nutrients intakes obtained from the two FFQ administered by the two interviewers. All analyses were performed with the SAS statistical package version 8.02 (SAS Institute, Cary, N.C., USA).

## Results

### Study 1: Validation

Women had a mean BMI of 25.8 ± 3.9 kg/m^2 ^and a mean age of 46.8 ± 7.9 y. Average daily nutrient intakes derived from FFQ and the 3-day food records are shown in Table 1. Total energy intake measured by the FFQ was not different from the intake assessed by the 3-day food record (difference below 5%) at week 0 of the dietary intervention (Table 1). For FFQ and the 3-day food record respectively, 34% (n = 24) and 38% (n = 27) of subjects had at baseline a ratio between energy intake and estimated basal metabolic rate at or below 1.35. Also, the intakes of proteins, CHO, SFA, PUFA, trans fatty acids, cholesterol, alcohol, vitamin C, folate, calcium and iron measured by the FFQ and by the 3-day food record at week 0 did not differ significantly. Measurement of total dietary fibers and soluble fibers differed significantly between the FFQ and the food-record but differences were below 10%. Mean nutrient intakes measured with FFQ were within 10% of values obtained with the 3-day food record for 14 of the 19 nutrients measured at week 0. For total lipids and MUFA the differences were significant but below 15%. Adjusting for energy intake did not alter these observations. Similar observations were noted at week 12 except for intakes of lipids, total dietary fibers and soluble fibers that were not anymore significantly different between the two methods and for PUFA intake that was estimated as being significantly higher with the FFQ (data not shown).

**Table 1:** Mean values of daily intakes of nutrients and Spearman correlation coefficients between values derived from the 3-day food record and the FFQ at week 0 of the nutritional intervention (n = 71).

	**Dietary record**	**FFQ**	**Difference (%)^a^**	**Unadjusted**	**Energy-adjusted**
**Energy (kcal)^b^**	2055 ± 521	2143 ± 568	4.3	0.29**	
**Protein (g)**	81.3 ± 16.4	82.6 ± 23.3	1.6	0.27*	0.36**
**CHO (g)**	245.0 ± 58.9	242.2 ± 60.5	-1.1	0.40**	0.60***
**Lipids (g)^b^**	80.1 ± 34.4	90.0 ± 34.8*	12.4	0.29*	0.56***
**SFA (g)^b^**	27.1 ± 14.0	30.0 ± 12.7	10.7	0.30**	0.56***
**MUFA (g)^b^**	33.9 ± 14.3	39.0 ± 16.3**	15.0	0.26*	0.48***
**PUFA (g)^b^**	13.1 ± 6.0	14.4 ± 7.0	9.9	0.38**	0.46***
**EPA (g)^b^**	0.06 ± 0.07	0.05 ± 0.04	-16.7	0.33**	0.33**
**DHA (g)^b^**	0.17 ± 0.23	0.12 ± 0.08	-29.4	0.30**	0.30**
**Trans fat (g)^b^**	3.4 ± 2.0	3.6 ± 2.6	5.9	0.45***	0.56***
**Cholesterol (mg)^b^**	280.1 ± 139.8	300 ± 116	7.5	0.30**	0.36**
**Dietary fiber (g)**	21.9 ± 6.4	19.7 ± 5.0*	-10.0	0.32**	0.38**
**Soluble fiber (g)**	7.3 ± 2.0	6.6 ± 1.7**	-9.6	0.22	0.27*
**Insoluble fiber (g)**	14.4 ± 4.7	13.2 ± 3.5	-8.3	0.32**	0.37**
**Alchool (g)^b^**	11.4 ± 11.2	10.9 ± 10.7	-4.4	0.61***	0.66***
**Vitamin C (mg)**	138.5 ± 57.3	137.0 ± 62.2	-1.1	0.19	0.19
**Folate (mcg)**	394.6 ± 115.4	383.2 ± 107.1	-2.9	0.32**	0.39**
**Calcium (mg)**	898.4 ± 320.5	962.3 ± 399.8	7.1	0.49***	0.56***
**Iron (mg)^b^**	15.1 ± 4.3	14.1 ± 4.1	-6.6	0.43**	0.53***

Value are means ± SD

^a ^(value derived from FFQ-value derived from 3-day food record)/(value derived from 3-day food record) × 100

^b ^Analyses were performed on log transformed values

Significant difference between the two methods *p ≤ 0.05; **p ≤ 0.01; ***p ≤ 0.0001

Correlations between FFQ and 3-day food record were statistically significant at * p ≤ 0.05; ** p ≤ 0.01; *** p ≤ 0.0001

Spearman correlations between values of nutrient intakes measured by the 3-day food record and those assessed by the FFQ at week 0 of the nutritional intervention are shown in Table 1. Analyses were performed on unadjusted as well as on energy-adjusted values. The average correlation coefficient for the nutrients presented in Table 1 was 0.44 at week 0 (energy-adjusted). Values derived from the FFQ and the 3-day food record were well correlated (energy-adjusted) for CHO, lipids, SFA, trans fatty acids, alcohol, calcium and iron (0.5 < r < 0.7) and moderately well correlated for proteins, MUFA, PUFA, cholesterol, dietary fibers, insoluble fibers, EPA and DHA (0.3 < r < 0.5). Further adjustment for age and BMI did not materially modify these correlations. Correlations between FFQ and dietary food record at week 12 were slightly higher than correlations at week 0 (data not shown).

Table 2 presents changes observed in response to the 12-week nutritional intervention derived from the 3-day food record and from the FFQ. For the majority of the nutrients analyses changes observed by the 3-day food record were similar to those observed by the FFQ except for vitamin C intake for which a higher increase was noted when dietary changes were assessed by the FFQ.

**Table 2:** Changes in daily energy and selected nutrients intakes derived from the 3-day food record and from the FFQ in response to the 12-week nutritional intervention (n = 71).

	**Dietary record**	**FFQ**
**Energy (kcal)**	-197 ± 464	-200 ± 500
**Protein (g)**	-1.4 ± 15.8	-0.5 ± 18.8
**CHO (g)**	-13.9 ± 60.8	-7.4 ± 59.2
**Lipids (g)**	-12.3 ± 32.5	-16.1 ± 28.3
**SFA (g)**	-6.2 ± 13.1	-9.6 ± 9.8
**MUFA (g)**	-4.2 ± 14.7	-4.7 ± 15.3
**PUFA (g)**	-1.1 ± 6.2	-0.8 ± 6.2
**EPA (g)**	0.06 ± 0.13	0.06 ± 0.06
**DHA (g)**	0.10 ± 0.40	0.11 ± 0.11
**Trans fat (g)**	-1.4 ± 2.1	-1.6 ± 2.4
**Cholesterol (mg)**	-51.9 ± 142.6	-65.8 ± 92.2
**Dietary fiber (g)**	3.0 ± 8.0	4.7 ± 6.9
**Soluble fiber (g)**	0.7 ± 2.7	1.2 ± 2.3
**Insoluble fiber (g)**	2.3 ± 5.7	3.5 ± 4.8
**Alcohol (g)**	-2.8 ± 10.4	-1.9 ± 9.4
**Vitamin C (mg)**	3.6 ± 65.9	29.4 ± 62.9*
**Folate (mcg)**	-7.8 ± 131.9	18.1 ± 113.8
**Calcium (mg)**	3.8 ± 304.1	33.6 ± 331.9
**Iron (mg)**	-0.3 ± 5.3	1.0 ± 4.3

Value are means ±

SD Significant difference between the two methods *p ≤ 0.05

Agreement between quartile classification of FFQ and 3-day food record is show in Table 3. Percentage of agreement varied from 29.4% for proteins to 64.7% for trans fatty acids for the lowest intakes (1^st ^tertile) and from 27.8% for CHO to 55.6% for alcohol for the highest intakes (4^th ^tertile). When considering all nutrients studied, it was found that, on average, 35.1% of subjects were categorized exactly in the same quartile by the FFQ and by the 3-day food record and 5.1% of the subjects were misclassified in extreme quartiles i.e. subject in the first quartile according to one method and in the fourth quartile according to the other (not shown).

**Table 3:** Percentage of agreement for the classification into quartiles of the distribution of selected dietary variables using either the 3-day food record or the FFQ at week 0.

	**Lowest quartile with 3-day food record and FFQ (%)**	**Highest quartile with 3-day food record and FFQ (%)**	**Exact agreement across quartiles (%)**
**Energy (kcal)**	47.1	38.9	39.4
**Protein (g)**	29.4	38.9	28.2
**CHO (g)**	41.2	27.8	26.8
**Lipids (g)**	35.3	38.9	33.8
**SFA (g)**	41.2	33.3	29.6
**MUFA (g)**	35.3	36.8	33.8
**PUFA (g)**	47.1	38.9	35.2
**Trans fat (g)**	64.7	38.9	47.9
**Cholesterol (mg)**	41.2	44.4	36.6
**Dietary fiber (g)**	47.1	44.4	31.0
**Alcohol (g)**	52.9	55.6	43.7

### Study 2: Reproducibility

The 32 subjects in study 2 had a mean BMI of 23.9 ± 3.6 kg/m^2 ^for women and 27.6 ± 5.1 kg/m^2 ^for men. The mean age was 42.5 ± 10.4 y for women and 41.2 ± 11.9 y for men.

Table 4 shows average daily nutrient intakes derived from the two FFQs (FFQ1, FFQ2) administered 4 weeks apart by two different dietitians. Measurement of total energy intake was not different between the two FFQs (difference of 6.8%). Also, the intakes of proteins, CHO, lipids, SFA, MUFA PUFA, trans fatty acids, cholesterol, alcohol and micronutrients measured by FFQ1 and by FFQ2 did not differ significantly. Adjustment for total energy intake did not alter these observations. Similar results were observed when analyses were performed within each gender separately. Subjects in the first group (in which dietitian #1 administered the 1^st ^FFQ) showed similar variation in nutrients intakes between FFQ1 and FFQ2 than subjects from the 2^nd ^group (in which dietitian #2 administered the first FFQ).

**Table 4:** Mean values of daily intakes of nutrients from two FFQ^a ^and Spearman correlation coefficients between values derived from the two FFQs^a^.

	**FFQ1**	**FFQ2**	**Difference (%)^c^**	**Unadjusted^d^**	**Energy-adjusted^d^**
**Energy (kcal)**	2283 ± 584	2128 ± 480	-6.8	0.73***	
**Protein (g)^b^**	89.4 ± 28.4	86.8 ± 24.4	-2.9	0.65***	0.83***
**CHO (g)**	286.3 ± 88.2	262.2 ± 80.6	-8.4	0.79***	0.66***
**Lipids (g)^b^**	84.6 ± 25.1	78.9 ± 19.8	-6.7	0.47*	0.82***
**SFA (g)^b^**	28.8 ± 10.1	27.1 ± 8.6	-5.9	0.51*	0.81***
**MUFA (g)^b^**	34.9 ± 11.0	32.6 ± 8.4	-6.6	0.52*	0.82***
**PUFA (g)^b^**	14.3 ± 6.2	13.1 ± 4.7	-8.4	0.60**	0.74***
**EPA (g)^b^**	0.06 ± 0.05	0.07 ± 0.06	16.7	0.55**	0.55**
**DHA (g)^b^**	0.12 ± 0.09	0.15 ± 0.12	25.0	0.55**	0.56**
**Trans fat (g)**	3.5 ± 1.7	3.2 ± 1.4	-8.6	0.60**	0.79***
**Cholesterol (mg)**	278.2 ± 102.8	269.3 ± 75.1	-3.2	0.47*	0.73***
**Dietary fiber (g)**	22.9 ± 7.9	20.8 ± 6.3	-9.2	0.76***	0.75***
**Soluble fiber (g)**	7.8 ± 2.4	7.1 ± 2.0	-9.0	0.82***	0.87***
**Insoluble fiber (g)**	15.0 ± 5.5	13.7 ± 4.4	-8.7	0.73***	0.81***
**Alchool (g)**	9.6 ± 6.7	9.4 ± 8.0	-2.1	0.76***	0.70***
**Vitamin C (mg)**	201.6 ± 103.5	165.2 ± 77.0	-18.1	0.81***	0.62***
**Folate (mcg)^b^**	442.0 ± 147.4	394.5 ± 128.5	-10.7	0.71***	0.69***
**Calcium (mg)^b^**	1153.5 ± 453.5	1085.1 ± 456.2	-5.9	0.79***	0.86***
**Iron (mg)^b^**	15.8 ± 5.7	14.5 ± 4.7	-8.2	0.64***	0.79***

Values are means ± SD

^a ^2 FFQ were administered 4 weeks apart

^b ^Student t test analyses were performed on log transformed values

^c ^(value derived from FFQ2 - value derived from FFQ1)/(value derived from FFQ1) × 100

^d ^Correlations between FFQ1 and FFQ2 were statistically significant at * p ≤ 0.05; ** p ≤ 0.01; *** p ≤ 0.0001

Table 4 presents Spearman correlations between nutrient intakes measured by FFQ1 and FFQ2. The average correlation coefficient for the nutrients presented in Table 4 is 0.74. Values derived from the two FFQs after adjustment for energy intake were generally very well correlated for proteins, lipids, SFA, MUFA, PUFA, trans fatty acids, cholesterol, dietary fibers, soluble and insoluble dietary fibers, alcohol, calcium and iron (0.7 < r < 0.9) and well correlated for CHO, EPA, DHA, vitamin C and folate (0.6 < r < 0.7). Partial correlations between nutrients derived from FFQ1 and those derived from FFQ2 were unchanged after adjusting for age, BMI and for the dietitian who administered the 1^st ^FFQ. In addition, similar correlations were observed when analyses were computed within each gender separately (not shown).

Table 5 shows agreement between quartile classification when FFQ1 was compared to FFQ2. Percentage of agreement varied from 28.6% for proteins to 75.0% for energy and alcohol for the lowest intakes (1^st ^quartile) and from 37.5% for SFA and MUFA to 75.0% for energy, proteins, CHO and alcohol for the highest intakes (4^th ^quartile). When considering all the nutrients studied, it was found that on average, 45.7% of subjects were categorized exactly in the same quartile by both FFQs and 2.6% of the subjects were misclassified in extreme quartile (not shown).

**Table 5:** Percentage of agreement for the classification into quartiles of the distribution of selected dietary variables using either FFQ1 or FFQ2.

	**Lowest quartile with FFQ1 and FFQ2 (%)**	**Highest quartile with FFQ1 and FFQ2 (%)**	**Exact agreement across quartiles (%)**
**Energy (kcal)**	75.0	75.0	62.5
**Protein (g)**	28.6	75.0	37.5
**CHO (g)**	50.0	75.0	59.4
**Lipids (g)**	50.0	62.5	40.6
**SFA (g)**	50.0	37.5	34.4
**MUFA (g)**	62.5	37.5	40.6
**PUFA (g)**	50.0	62.5	46.7
**Trans fat (g)**	62.5	50.0	40.6
**Cholesterol (mg)**	37.5	50.0	40.6
**Dietary fiber (g)**	62.5	50.0	43.8
**Alcohol (g)**	75.0	75.0	56.3

## Discussion

Accurate assessment of dietary intakes and dietary changes plays a central role in nutritional studies. Each tool used to evaluate dietary intakes has some strengths and limitations. Also, all standard dietary assessment methods are subjected to bias such as underreporting [6]. In our study, we developed a 91-items interviewer-administered FFQ that was sufficiently accurate to measure intakes of nutrients in the habitual diet of subjects from the Québec City metropolitan area and changes in nutrient intakes following a 12 week intervention promoting the Mediterranean food pattern.

In the validation study, coefficients of correlation between values derived from the FFQ and those obtained by the 3-day food record ranged from 0.30 to 0.60 for macronutrients and from 0.19 to 0.56 for micronutrients at week 0. It has been previously reported that correlation coefficients for validation studies ranged from 0.4 to 0.7, similar to our results after energy adjustment [6,15]. Also, our interviewer-administered FFQ did not significantly overestimate energy intake compared to a 3-day food record. The fact that the interviewer used food models to facilitate the estimation of portion size can contribute to explain this findings. It has been shown that FFQ can both under- and overestimate intakes of specific nutrients. In fact, many validation studies have reported that FFQ, as compared to food-record or 24-hour recall overestimate nutrients intakes as well as energy intake [16-20]. In contrast, other studies have reported that FFQ did not systematically overestimate energy and nutrients intakes [14,21-23].

Despite the fact that we obtained similar values for energy intake with both dietary methods, we can not exclude the possibility that both tools are subjected to underreporting and therefore underestimate usual dietary intakes. It has been previously suggested that subjects may tend to underreport actual food intake by as much as 20% when completing a weighted dietary record [24]. It has been argued that subjects who complete 3-day food record may change their nutritional food habits in order to simplify the recording of food intakes or to impress the dietitian. Also, errors in 3-day food records can be attributable to interpretation of the dietitian encoding the records. In our study, the same dietitian verified all the food records to make sure that dietary data were coded similarly for all participants. In the present study, 38% of subjects included in the validation study at week 0 had a ratio between energy intake to estimate BMR below 1.35. Considering that they had to be weight stable to be included in the study it is likely that these women were underestimating their habitual diet. Black et al concluded in a review that underreporting was observed in a great majority of nutritional surveys independently of the method used [25]. Earlier studies conducted in lean women demonstrated that underreporting was mainly explained by undereating [26] or underreporting snack foods [27] whereas in obese subjects underreporting could be explained by an underestimation in recording portion size and to social desirability. In addition, underreporting occurs more often among foods considered 'bad' or 'unhealthy' [28]. In our validation study, there were no significant differences between BMI of women who were considered as underreporters and women who did not underreport (not shown).

In a nutritional intervention, interpretation of the study outcomes with regard to dietary changes will depend not only of the validity and the reproducibility of the method used but also of the sensibility of the method to detect dietary changes in response to the intervention. In our nutritional intervention study, conducted in a sample of healthy women, both diet assessment methods detected similar dietary changes over the duration of the intervention. These findings suggested that our FFQ is sensitive to dietary changes in response to our intervention and could be used to assess dietary changes during a nutritional intervention. Our results are in agreement with study that showed that in response to a nutritional intervention a FFQ measured similar dietary changes as compared to 24-hour recalls [29] or 4-day food records [7].

The major differences between the two methods in our study were noted for total lipids and MUFA intakes. Our FFQ was designed to assess precisely lipid intake and many questions were asked about types of fat used to spread or to cook. The more important differences between FFQ and 3-day food record for MUFA and lipids could therefore be explained by the fact that it was difficult for participants to report precisely their lipid consumption when completing the FFQ. It has also been reported in obese men that underreporting of food record is usually specific to lipid intake [30] and it is thus possible that some women did not record all fats or foods high in fat consumed when completing their 3-day food record. Therefore, it is difficult to determine whether our FFQ tended to overestimate lipid or whether the 3-day food record tended to underestimate it. Also, dietary changes for these nutrients were in the same magnitude in response to our intervention with both methods. On the other hand, Mediterranean diet is usually considered high in MUFA. In North America, MUFA are mostly provided by partially hydrogenated vegetable oils and animals products [31]. In that context, MUFA to SFA ratio could be considered as a better indicator of a Mediterranean diet. In our study, we noted that this ratio was not different between the two methods at baseline and changes observed in response to the nutritional intervention did not differ significantly (not shown).

The agreement in quartile classification was acceptable for selected nutrients with a mean of 35.1% of subjects who were in exact agreement and 5.1% who were misclassified in extreme quartiles. This finding is similar to previous observations [16,19,32,33]. In many studies, classification in the same segment of the distribution using two different methods is found in 30% to 40% of subjects [16,32,34].

When analyses were performed at week 6 and 12 after the beginning of the nutritional intervention, coefficients of correlation were slightly higher than at week 0. We suggest that this finding be partly explained by the intervention effect. As previously reported [35] subjects could be influenced by a learning effect. In fact, subjects could be influenced by the first FFQ experience and be more adequately prepared for the second FFQ. The nutritional intervention may have also influenced the manner in which subjects were completing their 3-day food records during the study.

In a nutritional intervention, it is important to use a reproducible method to insure that dietary changes observed are due to the intervention effects and not to the instrument error. Our study suggests that the FFQ presents a good degree of reproducibility. In fact, in reproducibility studies the coefficients of correlation generally ranged from 0.5 to 0.7 [6]. In our study, coefficients of correlation ranged, after energy adjustment, from 0.62 for vitamin C to 0.83 for protein intakes. These values are similar to correlations reported by others [14,18,19,21,22,33,35-37].

In our reproducibility study, lower mean energy intake and nutrient intakes were found at the second administration of the FFQ as compared to the first FFQ (difference of approximately 10%). However, relatively high and uniform correlation coefficients for values derived from the two FFQs were observed. Riley et al [35] also reported with an administered FFQ that energy intake was 10% lower at the second FFQ administration and this reduction was uniform for all nutrients studied. In our study, intakes of most nutrients were systematically higher when measured with the first FFQ compared to the second one, except for alcohol consumption, which remained the same. Seasonal variation can not explain this difference because both FFQs were administered during the same season. The fact that subjects estimated a lower frequency of intake during the second administration of the FFQ may be explained by their earlier experience in completing the FFQ. Better general knowledge of dietary intakes could lead to a readjustment in estimation of intakes after the first administration of the FFQ and therefore changes in estimated energy intake.

## Conclusions

In conclusion, the FFQ that we developed to estimate usual average daily energy and nutrient intakes in subjects from the Québec City metropolitan area is valid and reproducible. Mean energy and nutrient intakes were estimated accurately by our FFQ compared to the 3-day food record. The fact that our FFQ was administered by a dietitian trained to insure a standardized administration of FFQ was important to optimize the validity and reproducibility of the method. We also showed that both methods appeared to underestimate energy intake in a large proportion of subjects. Usually, food records are considered as the gold standard method to assess dietary intakes. It is however important to recognize that food records also have their own limitations. In nutritional studies, an interviewer-administered FFQ, as the one we used in the present study, can be used to assess energy and nutrient intakes and requires less time to compute dietary informations than food records. FFQ also decreases the possibility of interpretation by the coding person. Finally, there is still a need to develop other efficient methods to measure dietary intakes that will permit to more closely match habitual dietary intakes of individuals in their living environment.

## List of abbreviations used

CHD: coronary heart disease

FFQ: Food Frequency Questionnaire

BMR: basal metabolic rate (BMR)

BMI: body mass index

NDS-R: Nutrition Data System for Research

CHO: carbohydrates

SFA: saturated fatty acids

MUFA: monounsaturated fatty acids

PUFA: polyunsaturated fatty acids

EPA: eicosapentaenoic acid

DHA: docosahexaenoic acid

## Competing interests

None declared.

## Authors' contributions

JG participated to data collection, performed data analysis and drafted the manuscript. GN and AL participated to data collection. BL and SL conceived the study, and participated in its design and coordination. All authors read and approved the final version of the manuscript.
